# Supplementary material for: Differential functional consequences of GRIN2A mutations associated with schizophrenia and neurodevelopmental disorders
Source: Sci Rep. 2024 Feb 2;14:2798. doi: 10.1038/s41598-024-53102-3 (PMC10837427; doi:10.1038/s41598-024-53102-3)
Supplement: Supplementary file 1 — Supplementary Figures. [file 41598_2024_53102_MOESM1_ESM.pdf]

Differential functional consequences of *GRIN2A* mutations associated with schizophrenia and neurodevelopmental disorders

Nate Shepard[1], David Baez-Nieto[1], Sumaiya Iqbal[2], Erkin Kurganov[1], Nikita Budnik[1], Arthur J. Campbell[1], Jen Q Pan[1], Morgan Sheng[1,3]*, Zohreh Farsi[1]*

[1]Stanley Center for Psychiatric Research, Broad Institute of MIT and Harvard, Cambridge, MA, USA

[2]The Center for the Development of Therapeutics, Broad Institute of MIT and Harvard, Cambridge, MA, USA

[3]Department of Brain and Cognitive Sciences, Massachusetts Institute of Technology, Cambridge, MA, USA

*Correspondence: msheng@broadinstitute.org and zfarsi@broadinstitute.org.

Figure S1

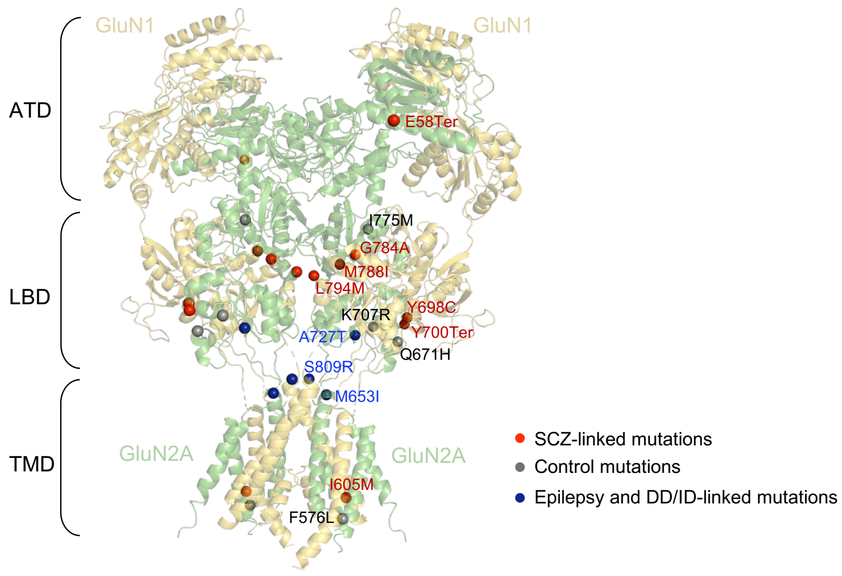
GluN1
GluN1
ATD
E58Ter
LBD
I775M
G784A
M788I
L794M
K707R
Y698C
A727T
Y700Ter
Q671H
S809R
M653I
TMD
GluN2A
GluN2A
I605M
F576L
SCZ-linked mutations
Control mutations
Epilepsy and DD/ID-linked mutations

Figure S1. *GRIN2A* mutations mapped on human NMDAR structure. Protein structure of human NMDAR (PDB ID 6IRH) with two GluN1 (light yellow) and two GluN2A (light green) subunits. *GRIN2A* mutations associated with SCZ (red) or epilepsy/DD/ID (blue) as well as control mutations (dark gray) are mapped on the two GluN2A subunits of human NMDAR. Two SCZ-associated missense mutations (Q811P, G591R), one control mutation (R586K), one DD/ID-linked mutation (L812M) as well as mutations within the CTD were not mapped due to no structure coverage.

Figure S2

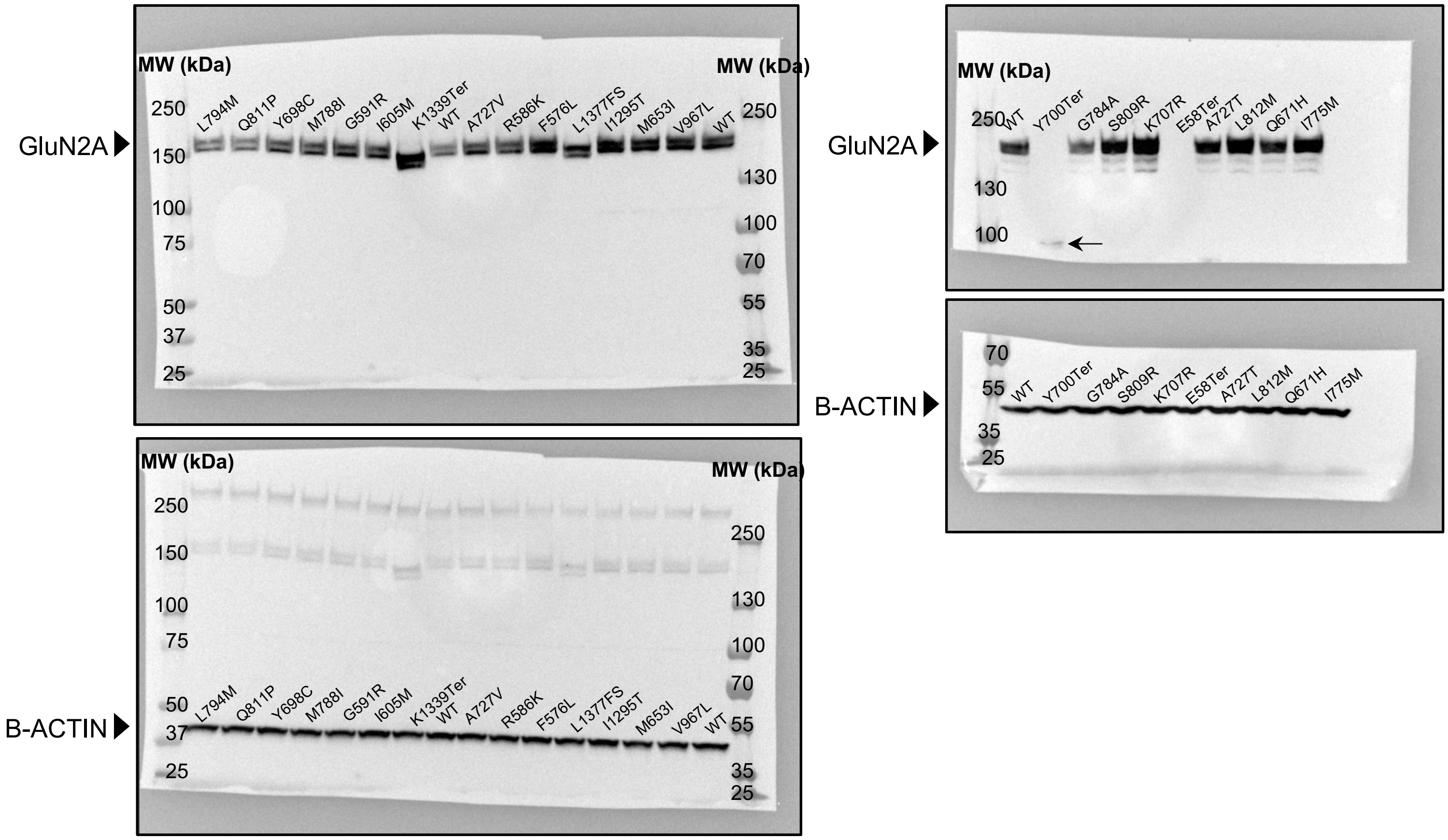
MW (kDa)
GluN2A
250
150
100
75
50
37
25
L794M
Q811P
Y698C
M788I
G591R
I605M
K1339Ter
WT
A727V
R586K
F576L
L1377FS
I1295T
M653I
V967L
WT
MW (kDa)
250
130
100
70
55
35
25
MW (kDa)
GluN2A
250
130
100
WT
Y700Ter
G784A
S809R
K707R
E58Ter
A727T
L812M
Q671H
I775M
B-ACTIN
70
55
35
25
WT
Y700Ter
G784A
S809R
K707R
E58Ter
A727T
L812M
Q671H
I775M
MW (kDa)
250
150
100
75
50
37
25
B-ACTIN
L794M
Q811P
Y698C
M788I
G591R
I605M
K1339Ter
WT
A727V
R586K
F576L
L1377FS
I1295T
M653I
V967L
WT
MW (kDa)
250
130
100
70
55
35
25

Figure S2. Uncropped western blot images of GluN2A variants. Western blots probing for GluN2A and β-ACTIN in lysates of HEK cells transiently transfected with GRIN1-GRIN2A constructs to express wild-type or mutant NMDARs. The arrow in Y700Ter lane indicates a faint band near the expected size for the Y700Ter fragment (~ 80 kDa).

Figure S3

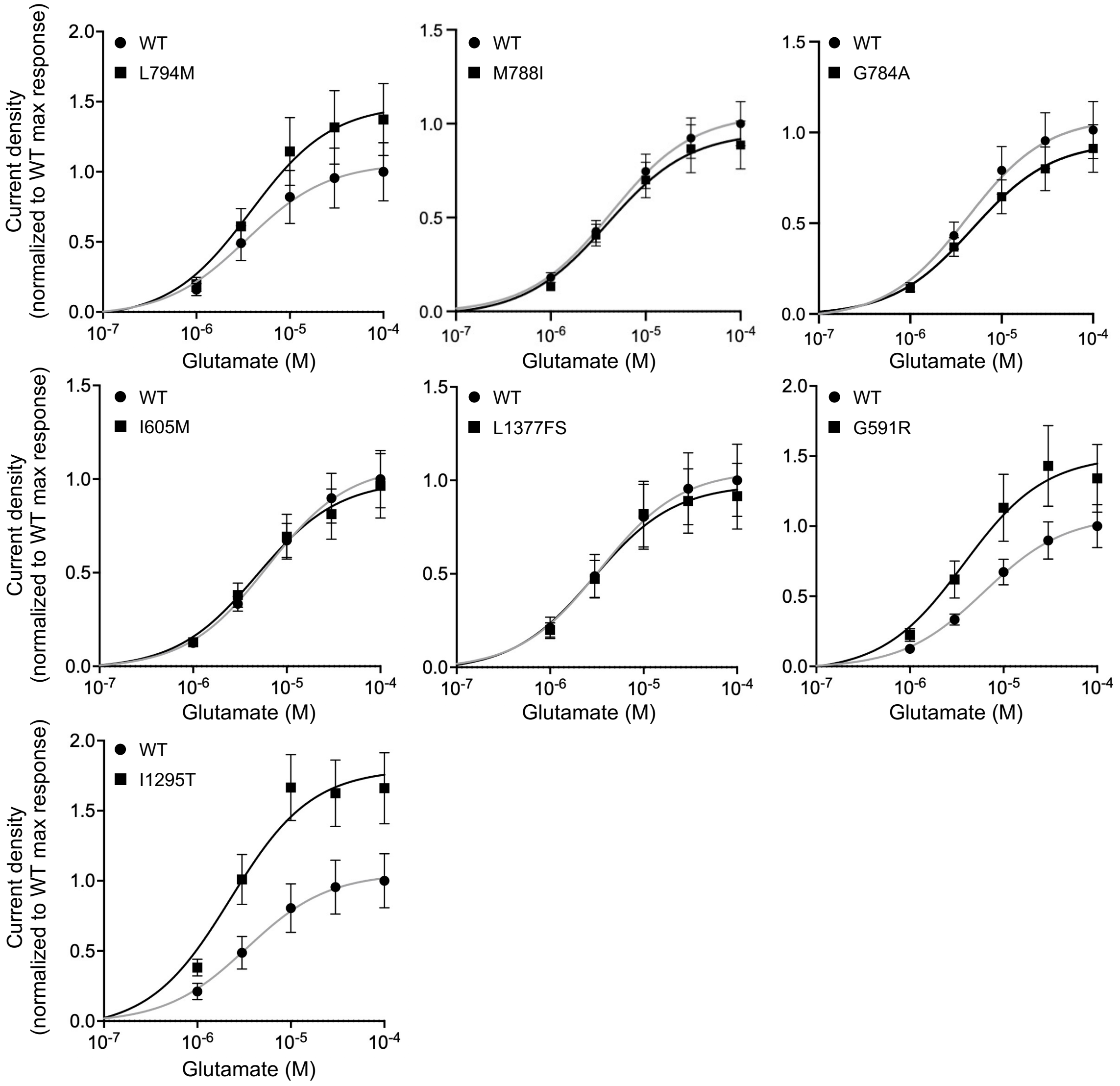
WT
L794M
M788I
G784A
I605M
L1377FS
G591R
I1295T
Current density (normalized to WT max response)
Glutamate (M)

Figure S3. Effect of SCZ-associated *GRIN2A* variants on NMDAR function. Averaged current density in response to increasing concentrations of glutamate in the constant presence of 30 μM glycine, normalized to maximal response, for NMDARs containing the wild-type or SCZ-associated GluN2A variants. The lines indicate a nonlinear regression three-parameter fit to each dataset. Data are displayed as mean ± SEM, n = 31-81; see Table 1 for number of cells recorded per variant.

Figure S4

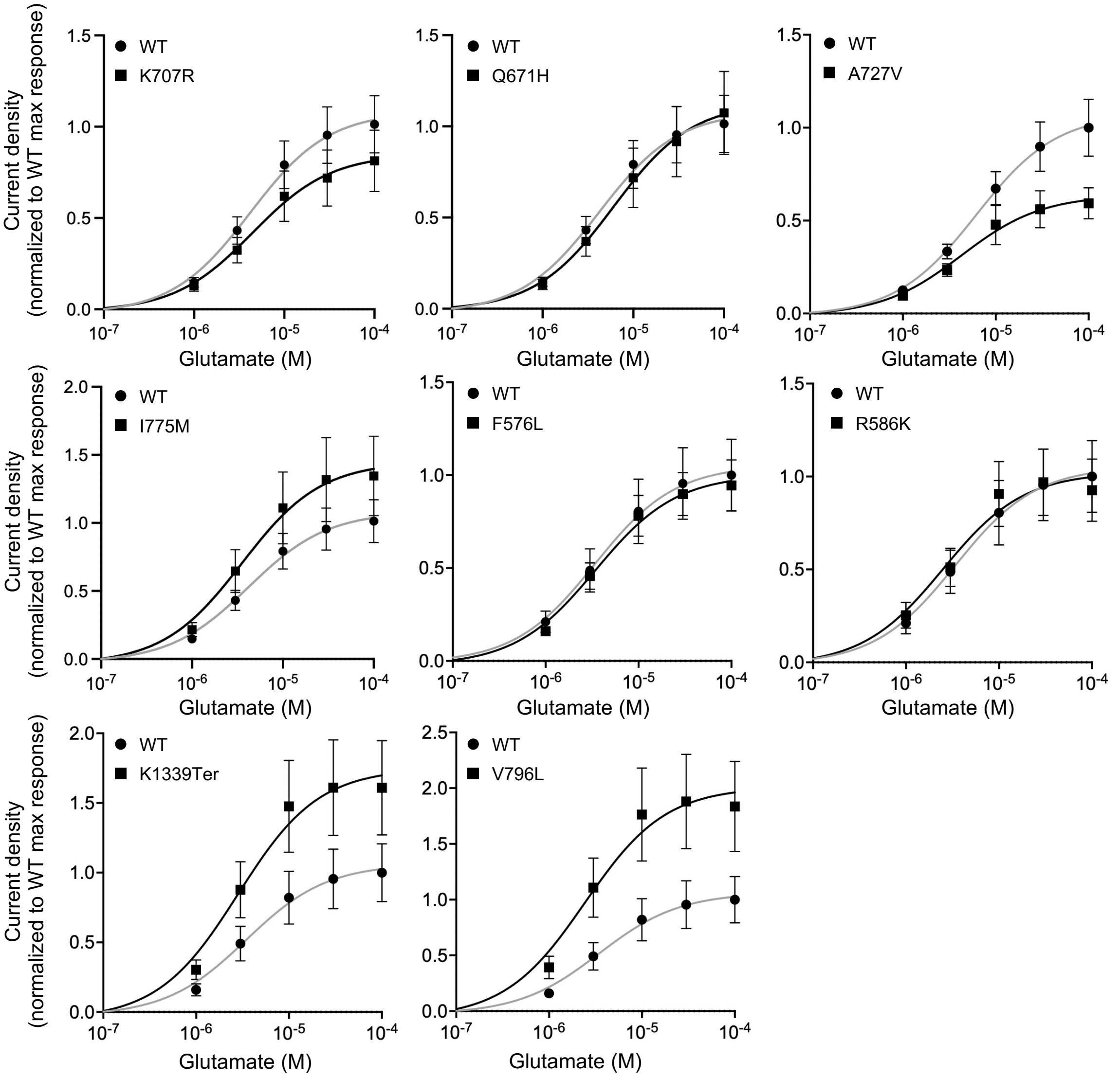
WT
K707R
Q671H
A727V
I775M
F576L
R586K
K1339Ter
V796L
Current density
(normalized to WT max response)
Glutamate (M)
10^{-7}
10^{-6}
10^{-5}
10^{-4}
0.0
0.5
1.0
1.5
2.0
2.5

Figure S4. Effect of non-pathogenic *GRIN2A* variants on NMDAR function. Averaged current density in response to increasing concentrations of glutamate in the constant presence of 30 μM glycine, normalized to maximal response, for NMDARs containing the wild-type or non-pathogenic GluN2A variants. The lines indicate a nonlinear regression three-parameter fit to each dataset. Data are displayed as mean ± SEM, n = 17-70; see Table 1 for number of cells recorded per variant.

Figure S5

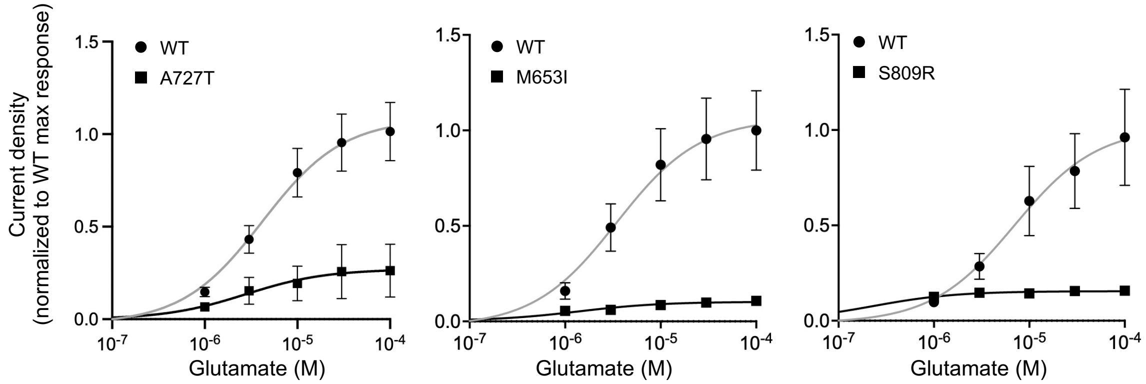

Current density
(normalized to WT max response)
1.5
1.0
0.5
0.0
WT
A727T
10-7
10-6
10-5
10-4
Glutamate (M)
WT
M653I
Glutamate (M)
WT
S809R
Glutamate (M)

Figure S5. Effect of Epilepsy and DD/ID-associated *GRIN2A* variants on NMDAR function. Averaged current density in response to increasing concentrations of glutamate in the constant presence of 30 μM glycine, normalized to maximal response, for NMDARs containing the wild-type or Epilepsy and DD/ID-linked GluN2A variants. The lines indicate a nonlinear regression three-parameter fit to each dataset. Data are displayed as mean ± SEM, n = 10-77; see Table 1 for number of cells recorded per variant.

Figure S6

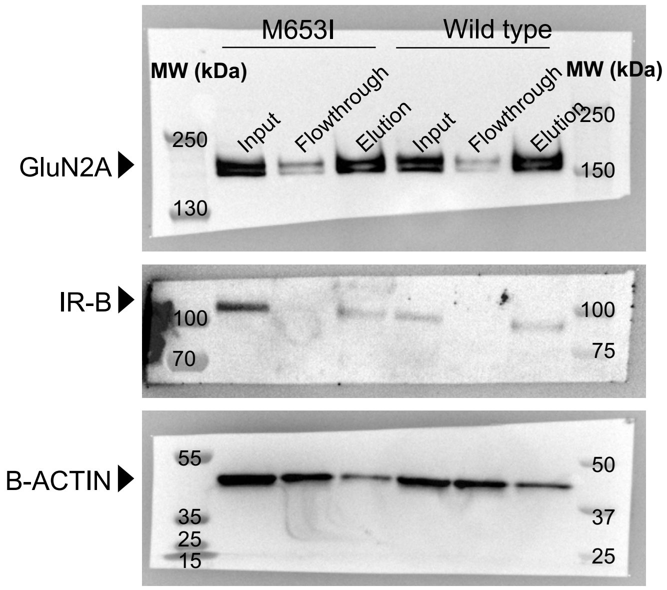
M653I
Wild type
MW (kDa)
MW (kDa)
Input
Flowthrough
Elution
Input
Flowthrough
Elution
250
150
250
130
GluN2A
IR-B
100
70
100
75
B-ACTIN
55
35
25
15
50
37
25

Figure S6. Original western blot images of the surface biotinylation experiment. Western blot probing for GluN2A, insulin receptor beta, and β-ACTIN in the input, flowthrough, and elution samples of surface biotinylation experiment done on HEK cells transiently transfected with *GRIN1-GRIN2A* constructs to express wild-type or M653I mutant NMDARs. Input, flowthrough, and elution represent total, internal, and surface expression respectively. IR-B: insulin receptor beta.
